# Metabolomics combined with molecular docking and dynamics simulation to investigate the mechanism of action of *Fibraurea recisa* Pierre in the treatment of chronic urticaria

**DOI:** 10.3389/fphar.2025.1571819

**Published:** 2025-05-21

**Authors:** Tian Xiao, Jie Tao, Jiaoyang Tan, Zhourong Zhao, Liping Yang, Chao Zhang, Xiaohua Duan

**Affiliations:** Yunnan Key Laboratory of Dai and Yi Medicines, Yunnan University of Chinese Medicine, Kunming, Yunnan, China

**Keywords:** network pharmacology, metabolomics, chronic urticaria, *Fibraurea recisa*, inflammation, immunity

## Abstract

**Background:**

This study investigated the mechanism of action of *Fibraurea recisa* Pierre (FRP) in the treatment of chronic urticaria (CU) using a rat model and combinatorial analysis of network pharmacology, metabolomics, and molecular dynamics and dynamics simulation data, providing a rationale for its clinical use.

**Methods:**

Twenty-four Sprague-Dawley rats were categorized into control, model, high-dose FRP (40 mg/kg body weight), and low-dose FRP (20 mg/kg body weight) groups. The CU model was induced by ovalbumin. Ultra-high performance liquid chromatography-tandem mass spectrometry (UPLC/MS) was used to estimate the levels of various components in FRP. The rats in different groups were evaluated for scratching behavior, histopathological changes in the skin tissues based on hematoxylin/eosin staining, and the levels of inflammatory factors and indicators of mast cell degranulation. Metabolomics, network pharmacology, molecular docking and dynamics simulation, and Western blotting were used to analyze the mechanism of action of FRP.

**Results:**

We identified 2,206 compounds in FRP based on UPLC/MS data analysis. Our data showed that the main active components in FRP were palmatine, jatrorrhizine, and coclaurine. FRP administration significantly reduced the scratching frequency, pathological characteristics of skin tissues, levels of inflammatory factors, and the degree of mast cell degranulation. Based on the combined analysis of metabolomics and network pharmacology data, phosphatidylinositol 3-kinase (PI3K)-protein kinase B (Akt) signaling pathway was identified as the key target of FRP. Molecular docking and molecular dynamics simulation demonstrated strong and stable binding of Akt with palmatine, jatrorrhizine, and coclaurine. Western blotting confirmed that FRP increased the levels of p-Akt and p-PI3K in skin tissue within the CU model.

**Conclusion:**

FRP significantly alleviated the symptoms and pathological changes of CU by modulating inflammation through upregulation of the PI3K-Akt signaling pathway.

## Graphical Abstract

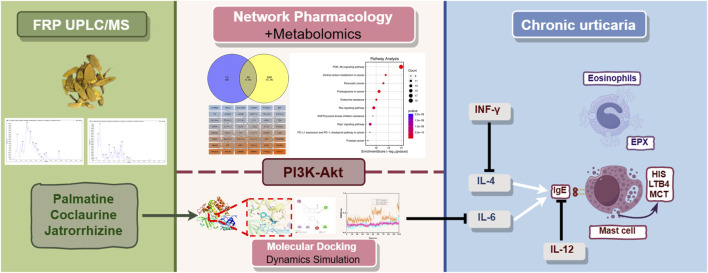

## 1 Introduction

Chronic urticaria (CU) is a common skin allergy with a global prevalence rate ranging from 0.1% to 3.4%. It is characterized by the presence of hives and itching for at least 6 weeks (Zhang et al., 2022). The pathogenesis of CU involves activation of mast cells and secretion of large amounts of inflammatory mediators such as histamine (HIS), leukotrienes (LT), and interleukins (IL), which trigger angioedema (swelling in the deeper layers of the skin) and recruit various immune cell types to the affected areas (Gimenez-Arnau et al., 2023). In the initial stages of CU, patients experience sudden itching and mild skin edema. As the disease progresses, the edema worsens and the wheals spread and merge. In severe cases, skin ulceration is observed. This disease negatively impacts the physical and mental health of the patients. More than half of the patients cannot recover spontaneously within 1 year. Therefore, there is an urgent need to discover new and effective interventions for this disease (He et al., 2021).

Currently, treatment with second-generation antihistamines is the primary treatment to control or relieve symptoms of CU (Valtellini et al., 2024). However, most patients do not respond adequately to conventional dosage of these drugs and higher dosage causes adverse reactions such as dizziness and drowsiness. In severe cases, patients may be treated with glucocorticoids. However, long-term use of glucocorticoids can lead to drug dependence and cause adverse effects, including centripetal obesity. Moreover, discontinuation of these drugs increases the risk of symptom rebound and exacerbation (Maurer et al., 2024).

Traditional Chinese medicine (TCM) is widely used for the clinical treatment of CU because of its high safety profile and a multi-component, multi-target approach leading to synergistic effects. TCM preparation from the dried vine stem of *Fibraurea recisa* Pierre (FRP) shows antibacterial and anti-inflammatory properties (Wang H. Y. et al., 2024). In traditional medicine practices of certain ethnic minorities, FRP is commonly used to treat skin diseases. In traditional Dai medicine, FRP is one of the main components in the classic formula “Yajie Shaba,” which is used for treating various allergies (Liping et al., 2022). Previous studies have confirmed that palmatine, an alkaloid in FRP, is effective in alleviating CU because of its anti-inflammatory and autophagy-regulating effects (Xiao et al., 2023). Although FRP is a promising drug for treating CU, the mechanisms of action of its various components have not well established.

Metabolomics and network pharmacology are valuable tools for studying complex diseases. They both have their own distinct advantages and disadvantages. Metabolomics is a high-throughput methodology to analyze the metabolome alterations in diseases, metabolite changes in response to drug treatment, and drug-disease relationships. However, metabolome data cannot be used to identify specific targets and mechanisms of drug action. Network pharmacology uses computational and experimental methods to identify the active components of drugs, predict disease targets, analyze drug target functions, and unravel the drug-disease target relationships. However, limitations in technology, data availability, and modeling approaches currently impact the accuracy of prediction results and require further improvements. The combined use of metabolomics and network pharmacology is a powerful approach for an in-depth analysis of drug action at the level of metabolites and systematic analysis of the drug-target interactions at the network level in human diseases (Shi et al., 2023; Li et al., 2023).

This study performed integrated network pharmacology and metabolomics analysis to determine the mechanisms by which FRP alleviates CU in a rat model. Firstly, UPLC/MS was used to determine the chemical composition of FRP. Then, network pharmacology was used to predict and analyze potential targets of action of each active component of FRP to determine their underlying mechanism in alleviating CU. Finally, metabolomics was used to determine the dynamic changes in the metabolome after treatment with FRP. Through this systematic research process, we aimed to determine the effects of FRP on the metabolic state, core targets, and main signaling pathways in the CU rats, and identify potential mechanisms by which FRP alleviates CU.

## 2 Material and methods

### 2.1 Experimental animals and drugs

24 male Sprague-Dawley rats of specific pathogen-free grade, aged 8 weeks and weighing approximately 200 ± 20 g, were used. These rats were provided by Changzhou Cavens Experimental Animals Co., Ltd., and the company’s experimental animal production license number is SCXK (Su) 2022-0010. The experimental rats were housed in an SPF-grade environment. The breeding temperature was strictly controlled within the range of 18°C–25°C, the relative humidity was maintained at 40%–60%, the light and dark periods were each 12 h and alternated, and the experimental rats had free access to food and water. All operations and research contents involving experimental animals have been approved by the Animal Ethics Committee of Yunnan University of Chinese Medicine (Grant No.: R-062022157).

Loratadine (HY-17043, MCE, Shanghai, China). Ovalbumin (OVA, S7951, Sigma Aldrich, St. Louis, MO, United States). Palmatine, coclaurine, jatrorrhizine (AB1049, AB0718, AB0536, Chengdu Alpha Biotechnology Co., Ltd.) FRP was purchased from Guangxi Suli Medicinal Materials Co., Ltd., and was identified by Yin Zili, associate professor of Yunnan University of Chinese Medicine. Weigh 480 g of FRP, decoct it with eight times the volume of distilled water for the first time and six times the volume of distilled water for the second time, and each decoction lasts for 30 min. Remove the impurities and retain the obtained water extract of the medicine for further processing.

### 2.2 UPLC/MS for the identification of FRP components

100 μL of FRP was added to a centrifuge tube, 100 μL of 70% methanol (Merch, Darmstadt, Germany) was added, and vortex for 15 min. 12,000 r/min, 4°C, centrifugation for 3 min, pipetting the supernatant, filtering with microporous filter membrane and then detecting on the machine. A is ultrapure water, and B is acetonitrile (Merch, Darmstadt, Germany). The flow rate was 0.35 mL/min, the column temperature was 40°C, and the injection volume was 2 μL. The elution gradient program was set to: Elution gradient: 0 min 5% B, 95% B in 9 min, and maintained at 95% for 1 min, 10.00–11.10 min, reduced to 5% B, and equilibrated at 5% to 14 min. The mass spectrometry conditions were set to electrospray ion source temperature 550°C; Ion spray voltage: 5,500 V (positive ion mode), 1–4,500 V (negative ion mode); The ion source gas I, gas II, and curtain gas are set to 50, 60, and 25 psi, respectively. Based on the self-managed Metware Database, the FRP component is obtained by using the CV value of <0.5 as the filter condition.

### 2.3 Determination of the content of effective components in FRP

Take 2 mL of the FRP, add 25 mL of acetonitrile and 0.1% phosphoric acid (P816338, Shanghai Macklin Biochemical Technology Co., Ltd.) in a ratio of 1:1, perform ultrasonic treatment at 37°C for 40 min (power: 250 W, frequency: 360 kHz). Let it cool down to room temperature, make up the volume to 25 mL with acetonitrile and 0.1% phosphoric acid, and filter it through a 0.45 μm filter membrane before injecting it into the machine. According to the literature, the main active components in FRP are palmatine (AB1049, Chengdu Alpha Biotechnology Co., Ltd., Chengdu, China), jatrorrhizine (AB0536, Chengdu Alpha Biotechnology Co., Ltd., Chengdu, China), and coclaurine (AB0718, Chengdu Alpha Biotechnology Co., Ltd., Chengdu, China). Therefore, accurately weigh 5 mg of palmatine, jatrorrhizine, and coclaurine reference substances respectively, place them in a 100 mL volumetric flask, add acetonitrile and 0.1% phosphoric acid in a ratio of 1:1 to completely dissolve them. Finally, obtain a solution containing 50 μg of the reference substances per 1 mL. Shake well and filter it through a 0.45 μm microporous filter membrane before injecting it into the machine (Agilent 1260 Infinity III Liquid Chromatography System, Agilent Technologies, Inc., Santa Clara, United States).

The model of the chromatographic column is Agilent 5 TC-C18 (Gimenez-Arnau et al., 2023) 250 × 4.6 mm (article number: 588925-902). Use acetonitrile (A) and 0.1% phosphoric acid aqueous solution (B) as the mobile phase. The gradient elution program is set as follows: 0–5 min, 20%–50% A; 5–10 min, 50%–80% A; 10–20 min, 80%–100% A. The flow rate is set at 1 mL/min, the column temperature is set at 25°C, the detection wavelength is 265 nm, and the injection volume is 10 μL.

### 2.4 Group administration and model replication of CSU

SD rats were divided into control group, model group, FRP high-dose group (FRP-H) and FRP low-dose group (FRP-L). Except for the control group, other rats were intraperitoneally injected with 1 mg of OVA (S7951, Sigma Aldrich, St. Louis, MO, United States) suspension on days 0, 2, and 4, and intraperitoneally injected with 2 mg of OVA on day 14. On days 5–14 after modeling, the control group and the model group were gavaged with an equal volume of distilled water once a day. The concentrations of FRP-H and FRP-L groups were 40 mg/kg and 20 mg/kg.

### 2.5 Rat scratching behavior

Rats exhibit scratching behavior when they feel itching, and we assessed the relief of itching in rats by scratching behavior. When 10 min after the last intraperitoneal injection of OVA intervention, the scratching frequency of rats in each group was evaluated within 20 min. The scratching behavior is judged by the rats rubbing the skin on their backs, scratching their heads and torsos, and rubbing against each other.

### 2.6 Histopathological detection of rat skin

The skin tissue of rats in each group was fixed with 4% paraformaldehyde at 4°C for 12 h, embedded with paraffin wax and cut into 5 μm thick sections, deparaffinized and hydrated. Stain with hematoxylin eosin (HE, KGA224, KeyGEN Biotech, NanJing, China) for 5 min at room temperature to study pathological changes, dehydrate with ethanol gradient, mount with neutral gum, and observe and photograph under a light microscope with a field of view of 100×. The remaining slices are stored at room temperature for subsequent experiments.

### 2.7 Detection of the expression of inflammatory factors in skin tissue

After the blood was taken, the rats were euthanized by cervical dislocation, and the shaved back skin tissue was obtained for subsequent experimental analysis. After blood collection, shaved back skin tissue was obtained for subsequent experimental analysis. The skin tissue of each group was washed with pre-cooled PBS at 4°C, weighed and minced, and normal saline was added at a ratio of 1:9 to make skin tissue homogenate. Centrifuge at 5,000 *g* for 10 min, take the supernatant for detection, and detect the expression of IL-4 (SEKR-0004, Solarbio, Beijing, China), IL-6 (SEKR-0005), IL-12 (E-EL-R0064c; Elabscience, TX, United States) and Interferon-γ (IFN-γ, E-EL-R0009, Elabscience) in skin tissues according to the instructions of the ELISA kit.

### 2.8 Mast cell activation and degranulation detection

Toluidine blue staining is commonly used for skin mast cell testing. The prepared skin tissue wax tablets were soaked in 0.5% toluidine blue solution (G1032, Servicebio, Wuhan, China) for 30 min at room temperature and washed with distilled water. Soak in 0.5% glacial acetic acid solution for 5 s and dehydrate with ethanol and transparent xylene. It is capsulated with neutral gum. Finally, the light microscope (Olympus Corporation, Japan) was randomly selected for 100 × without repeating, and mast cell counts were taken.

### 2.9 Mast cell tryptase (MCT) and eosinophil protein X (EPX) assays

After the wax tablets of skin tissue were dewaxed, the antigen was extracted in 0.01 mol/L citrate, and the antigen was exposed in the microwave for 20 min 0.03% H_2_O_2_ was added to inactivate endogenous peroxidase for 15 min, and blocked with goat serum blocking solution for 20 min at room temperature. Subsequently, sections are mixed with anti-TPSAB1 antibody (13343-1-AP, Proteintech, Wuhan, China) at a 1:50 dilution and with anti-EPX antibody (bs-3881R) (Bioss, Beijing, China) at a 1:100 dilution and incubated overnight at 4°C. After rewarming at 37°C for 1 h, incubate with secondary antibody and biotin-labeled goat anti-rabbit IgG for 30 min, then continue with biotin-labeled streptavidin for 30 min. t is chromogenic with the DAB kit, counterstained with hematoxylin, and sealed with neutral resin. Hooting at 200 levels × random optical microscope (Olympus Corporation, Japan) was not repeated, and the Image-Pro Plus 6.0 software detected the comprehensive optical density value for each field of view.

### 2.10 Detection of biochemical markers in serum

Rats in each group gently pressed the sides of the orbit with their fingers. After protruding the eyeball, use a capillary to enter through the gap between the eyeball and the corner of the eye. A blood sample can be collected by gently rotating the capillary and slowly moving it behind the eyeball. Centrifuge the collected blood sample at 3,000 r/min for 10 min, aspirate 1 mL of supernatant, and follow the instructions of the ELISA kit. The levels of immunoglobulin E (IgE, SEKR-0019), LTB4 (CSB-E08035r, Cusabio, Wuhan, China), and HIS (E-EL-0032c) in serum were detected at a wavelength of 450 nm using a microplate reader.

### 2.11 Metabolomics

50 μL of rat plasma in the model group and FPR-H group are removed and 200 μL of methanol solution containing internal standard is added. Hake for 5 min, centrifuge at 18,000 rpm for 10 min, and take 200 μL of supernatant. After evaporation by vacuum evaporator, 100 μL of pure water and 100 μL of methanol are reconstituted. Centrifuge at 18,000 rpm for 10 min, and finally take 80 μL of supernatant for storage. 5 μL was filtered through a 0.22 μm filter membrane for LC-QTOF/MS (Sciex TripleTOF 5600+ LC-QTOF/MS, Sciex) for positive and negative ion detection, and the detection results were compared between groups, and the results were repeated twice. Liquid phase conditions: Waters HSS T3 1.8 μm, 2.1 × 100 mm Column with 0.3 mL/min flow rate, 40°C column heater, 5 μL injection volume, Aqueous phase A (ultrapure water + 0.1% formic acid), B phase organic phase (acetonitrile), gradient elution program is: 0–1.5 min, 95% A, 5% B; 1.5–2.5 min, 95%–85% A, 5%–15% B; 2.5–6 min, 85%–40% A, 15%–60% B; 6–10 min,40%–5% A,60%–95% B; 10–12 min,5% A,95% B; 12–12.5 min, 5%–95% A, 95%–5% B; 12.5–15.5 min, 95% A, 5% B. Mass spectrometry conditions: The positive and negative ion modes were scanned by Turbo V electrospray ionization, and the parameter was set to the ion spray voltage was 7 kV; Turbine spray temperature, 550°C; Declustering potential, 70 V; Collision energy, 30 eV. The TOF/MS has a scanning range of m/z 50–1,200 Da.

### 2.12 Network pharmacology

The chemical components of FRP detected by UPLC/MS were screened out for network pharmacology analysis. The screening conditions were set as high for gastrointestinal absorption, yes for BBB permeability, and at least two yes for drug similarity, and the active ingredient of FRP was used as the product if it met the criteria. The Smiles number of the compound was searched in the Pubchem database, and the target of the compound was predicted by the Swiss target perdiction database, and the target with “Probability” greater than 0.1 was selected for subsequent analysis. With “CSU” as the keyword, the gene name was transformed by the OMIM, TTD and Genecards databases, and the duplicate values were deleted as the target of CSU. The intersections of the screened active ingredient targets and disease targets were made, and the intersection results were imported into Cytospace 3.9.1 software, and the network diagram of “FR-active ingredient-target” was drawn, and the main active ingredients of FRP in the treatment of CSU were screened out according to the degree value. Import the intersection targets into the String database, set the minimum interaction score to 0.4, check the hidden protein name, and output it in TSV (A-B) format. Cytoscape 3.9.1 software was imported, the network diagram was drawn, and the key targets of Rhubarb vine in the treatment of CSU were screened according to the degree value. Finally, the intersection targets were introduced into the micro-biotech data analysis platform for GO enrichment analysis and KEGG enrichment analysis to find the main pathways for FRP to intervene in CSU. Using the key targets as receptors and the components as ligands for molecular docking, select the top three components and targets ranked by binding energy. Use the Gromacs program to perform molecular dynamics simulations on the protein-small molecule complexes to evaluate the dynamic laws of the binding between ligands and receptors.

### 2.13 Detection of Akt, p-Akt, P13K, p-P13K protein expression

The skin tissue in each group was washed with pre-cooled PBS, weighed and shredded. Add 1 mL of RIPA lysate containing PMSF per (Solon, OH, United States) 100 g of tissue, centrifuge at 12,000 g at 4°C for 5 min, and transfer the supernatant to a pre-chilled EP tube. Protein quantification was performed according to the BCA kit method. Depending on the molecular weight of the protein to be measured, SDS-PAGE gels are prepared. Add an appropriate amount of pre-cooled 1 × of running buffer, and add the sample and the reference protein to the lane. Adjust the voltage to bring the destination strip to the predetermined position. After shearing the PVDF (Millipore, Schwalbach, Germany) membrane and transferring the membrane at constant pressure, the membrane was stained with Ponceau red s staining solution for 5 min. Put it in an antibody incubation box containing TBST, rinse it on a shaker for 5 min, discard TBST, and add 5% skimmed milk powder prepared with TBST. Block at room temperature shaker for 1 h, discard the blocking solution, add diluted primary antibody (Akt antibody dilution at a ratio of 1:500 (ab8805, Abcam, Cambridge, United Kingdom), p-Akt at a ratio of 1:1,000 (ab38449, Abcam, Cambridge, United Kingdom), P13K at a ratio of 1:20,000 (ab191606, Abcam, Cambridge, United Kingdom), p-P13K (ab182651, Abcam, Cambridge, United Kingdom) at a ratio of 1:500, anti-β-actin-antibody at a ratio of 1:1,000 (ab8229, Abcam, Cambridge, United Kingdom), and incubate overnight at 4°C. Wash the membrane five times with TBST for 5 min each time. Continue to add diluted secondary antibodies (Rabbit Anti-Mouse IgG H&L (HRP) (ab6728, Abcam, Cambridge, United Kingdom) diluted 1:10,000 and Goat Anti-Rabbit IgG H&L (HRP) (ab6721, Abcam, Cambridge, United Kingdom) diluted 1:10,000 and incubated for 1 h at room temperature. Wash the membrane five times with TBST for 5 min each time. After soaking in liquid A and solution B of the prepared luminescence reagents for 5 min, the detection was carried out with Tanon 6600 luminescence imaging workstation (Tanon 6600 Luminous Imaging Workstation, Tanon Corporation). The optical density value was analyzed by Image Pro Plus 6.0 software, and the relative expression level of the protein was calculated as the gray value of the target protein/the gray value of the internal reference protein.

### 2.14 Data analysis

Data were analyzed and graphed using Graphpad Prism 9 (Version 9.4.0). All data were presented as means ± SD, and statistical differences between groups were performed using one-way ANOVA and Tukey’s tests. P value of less than 0.05 was considered to be significant.

## 3 Results

### 3.1 Chemical composition of FRP based on UPLC/MS

**FIGURE 1:**
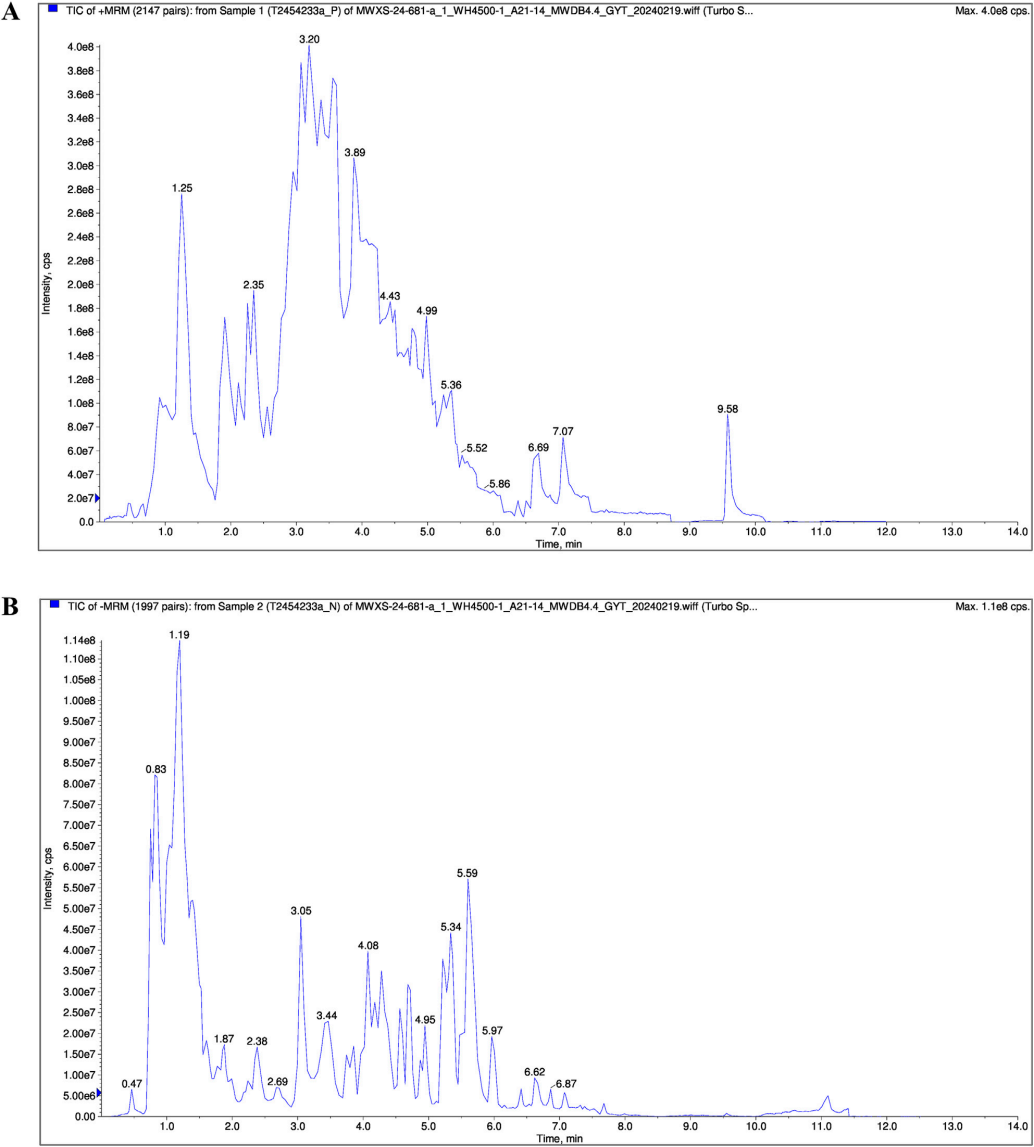
The total ion chromatogram of the FRP sample in **(A)** positive and **(B)** negative ion mode.

The chemical composition of FRP was analyzed by UPLC/MS. The total ion chromatogram of the FRP sample recorded in both positive and negative ion modes is shown in Figure 1. Based on the UPLC/MS results, we identified 2,206 compounds in FRP, including 452 alkaloids, 435 amino acids and their derivatives, 288 phenolic acids, 146 lignin and coumarin compounds, 145 organic acids, and 134 terpenoids. The top 1% of metabolites are in Table 1.

**TABLE 1:** The compounds with a content ranking in the top 1%.

Compounds	CAS
13,13α-Didehydro-9,10-dimethoxy-2,3-(methylenedioxy)-berbine	
1-[(4-hydroxyphenyl) methyl]-7-methoxy-1,2,3,4-tetrahydroisoquinolin-8-ol	
Isocorybulbine	22672-74-8
Corybulbine	518-77-4
Jatrorrhizine	3621-38-3
Coclaurine	486-39-5
3,4,11-trimethoxy-13-methyl-7,8,12b,13-tetrahydro-5h-6-azatetraphen-10-ol	
Tetrahydroprotopapaverine	26193-25-9
Reticuline	485-19-8
Dehassiline	142717-64-4
O-Rnethylstepharinosine	
Lauroscholtzine; N-Methyllaurotetanine	2169-44-0
N (6), N (6)-Dimethyl-L-lysine	2259-86-1
Dehydrocrebanine	77784-22-6
Columbamine	3621-36-1
Adenine	73-24-5
Zarzissine	160568-14-9
Vidarabine	5536-17-4
Dehydrocorydalmine	6877-27-6
Adenosine	58-61-7
9-Alpha-Ribofuranosyladenine	
9-Arabinosyladenine	
Palmatine	3486-67-7

### 3.2 Determination of active components in FRP

**FIGURE 2:**
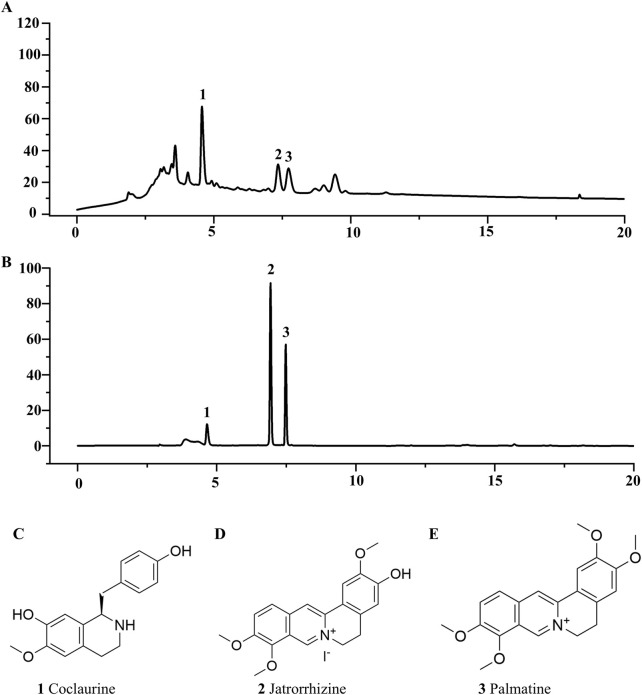
The liquid chromatograms of **(A)** the test sample and **(B)** the mixed reference substances of Coclaurine, Jatrorrhizine, and Palmatine. **(C)** The chemical structures of Coclaurine, **(D)** Jatrorrhizine, and **(E)** Palmatine.

Contemporary phytochemical studies have reported that benzylisoquinoline alkaloids are the principal bioactive constituents in FRP. Systematic network pharmacology screening data demonstrated that palmatine, coclaurine and jatrorrhizine were the primary active alkaloids in FRP. At 265 nm, retention times for the FRP test samples and coclaurine were 4.568 min and 4.621 min respectively; retention times for the FRP test samples and jatrorrhizine were 6.925 min and 7.347 min, respectively; retention times for the FRP test samples and palmatine were 7.471 min and 7.727 min, respectively (Figure 2). The separation method showed good specificity because the differences in retention times between the reference compounds and the test samples were all within ± 0.5 min.

### 3.3 Effect of FRP on scratching behavior in rats

**FIGURE 3:**
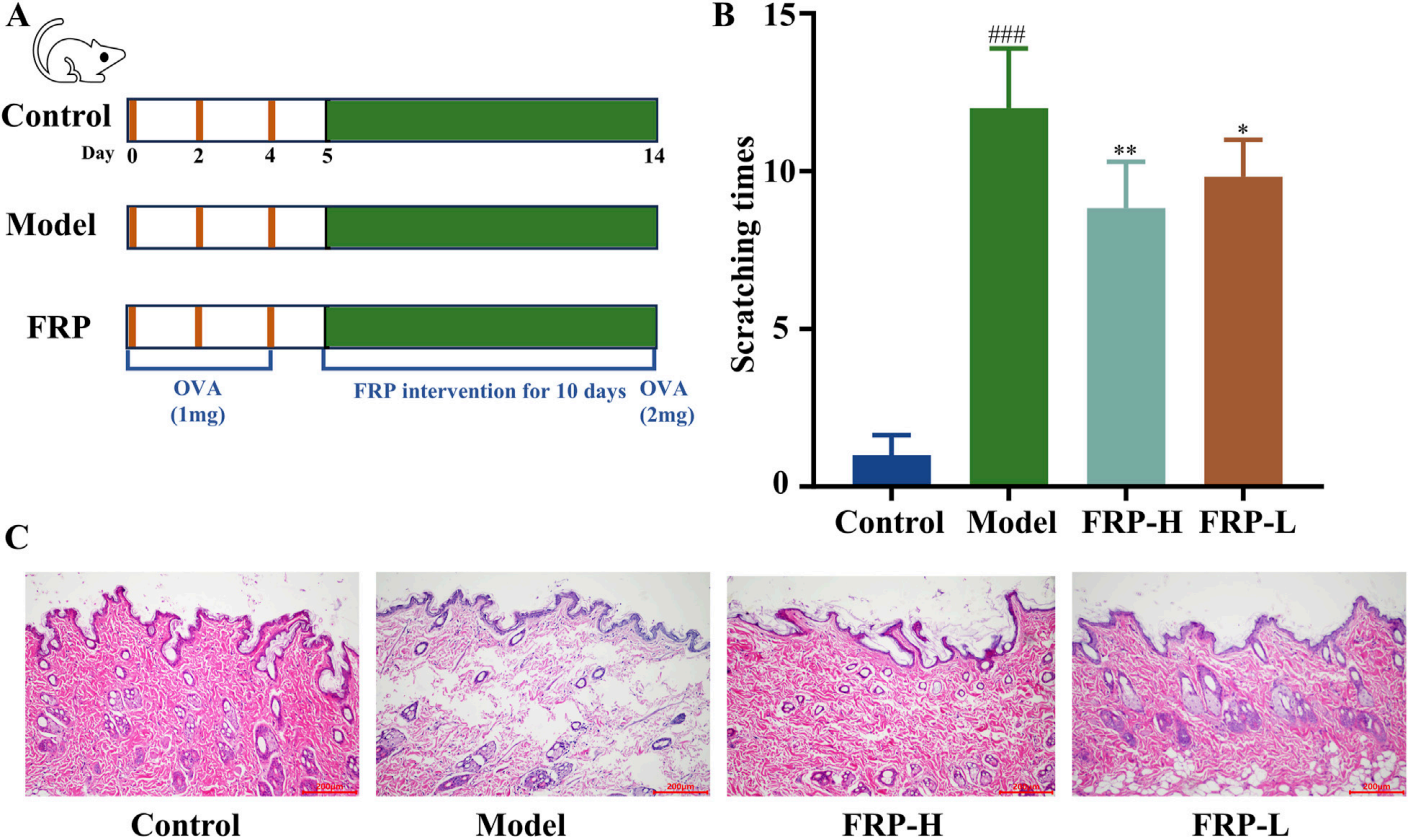
**(A)** The experimental procedure. **(B)** The effect of FRP on the scratching frequency of rats. **(C)** The effect of FRP on the pathological changes of rat skin tissues. All data are presented as $\bar{X}$ ±SD deviation (n = 6). Compared with the Control group, ^###^P < 0.001; compared with the model group, ^*^P < 0.05, ^**^P < 0.01.

As shown in Figure 3A, we generated the CU model rats and the FRP-treated CU model rats. Compared with the control group, rats in the CU model group and the FRP-treated CU model group showed different degrees of itching response (P < 0.001). The FRP-H and FRP-L groups showed significant improvements in scratching latency and time compared to the CU model group (P < 0.01 and P < 0.05, respectively; Figure 3B).

### 3.4 Effect of FRP on the histopathological changes in the rat skin tissues

According to the H&E staining results, the skin of rats in the control group showed normal epidermal structure, including uniform thickness, neat and orderly arrangement of cells, consistent staining, and normal hair follicle structure without significant dilation of the capillaries. In the CU model group, the epidermal structure was partially absent because of parakeratinization or hyperkeratosis, and the granular layer was thickened. Furthermore, we observed disordered collagen fibers, widened fiber bundle distance, dilated blood vessels and lymphatic vessels, and presence of multiple inflammatory cell infiltrates around superficial blood vessels. However, FRP intervention significantly improved CU-related histopathological characteristics (Figure 3C).

### 3.5 Effect of FRP on the levels of inflammatory cytokines in rats

**FIGURE 4:**
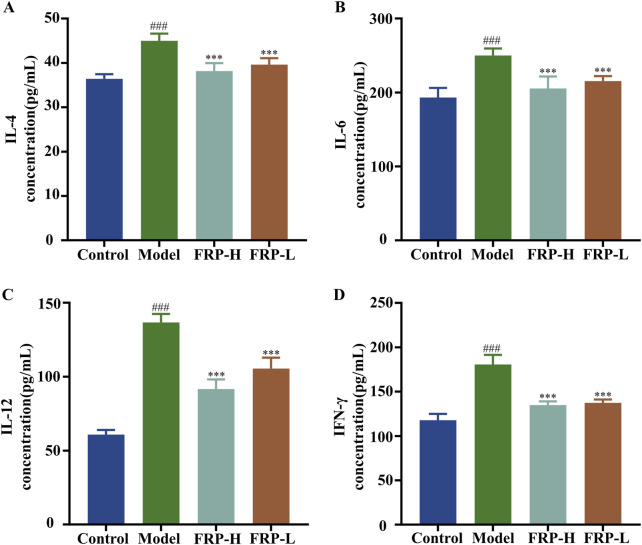
The effects of FRP on the levels of inflammatory factors **(A)** IL-4, **(B)** IL-6, **(C)** IL-12, and **(D)** IFN-γ in the skin tissues of rats in each group. All data are presented as $\bar{X}$ ± SD deviation (n = 6). Compared with the Control group, ^###^P < 0.001; compared with the model group, ^***^P < 0.001.

The levels of IL-4, IL-6, IL-12, and IFN-γ in skin tissue were significantly higher in the model group compared with the control group (P < 0.001), but were significantly decreased after FRP intervention (P < 0.001; Figure 4). These results suggested that FRP treatment suppressed the release of inflammatory mediators in CU.

### 3.6 Effect of FRP on the skin mast cells in rats

**FIGURE 5:**
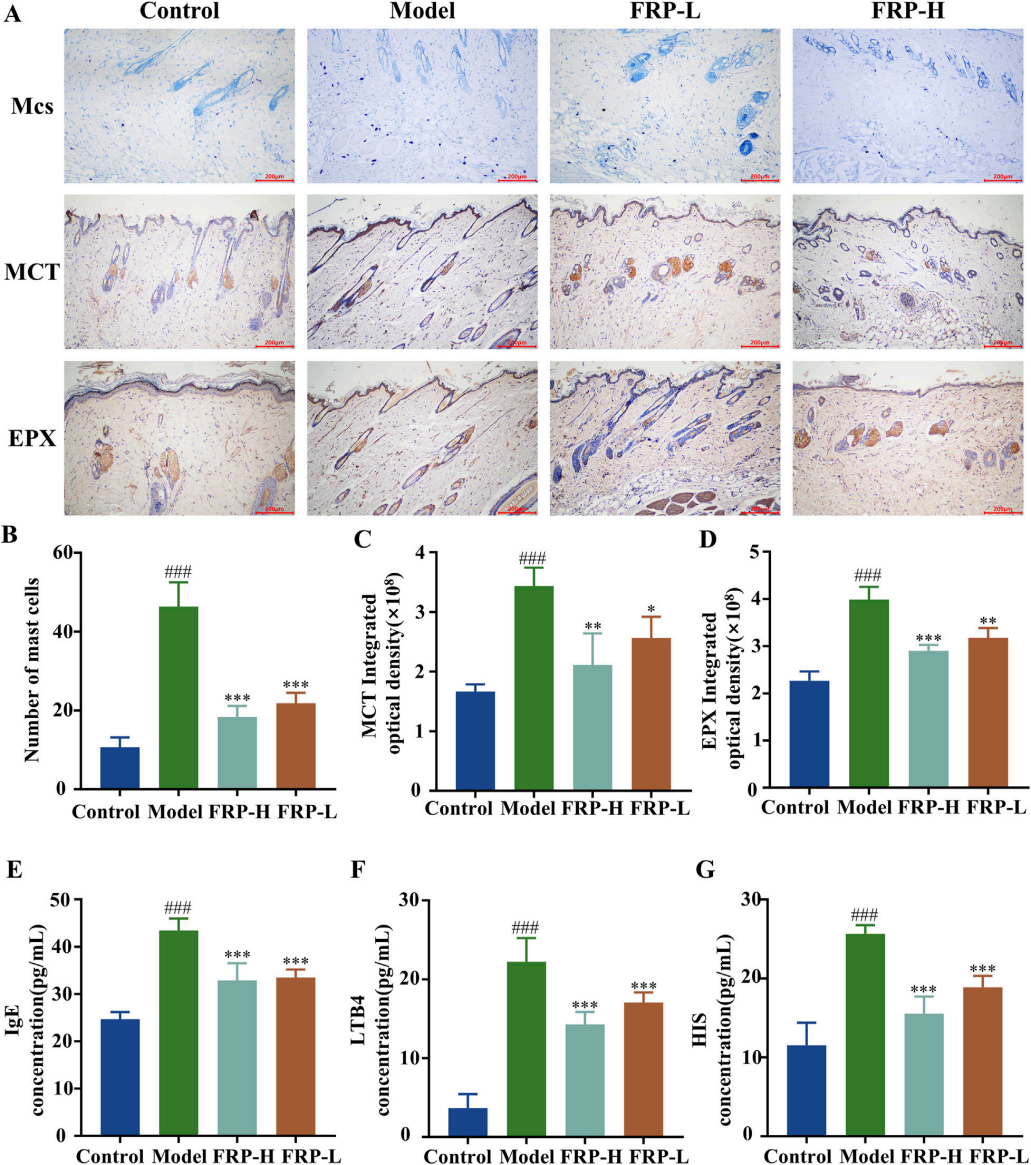
**(A)** Immunohistochemical images of mast cells, MCT, and EPX in rats of each group. The effects of FRP on **(B)** the number of mast cells, **(C)** the expression of MCT, and **(D)** the expression of EPX in rats. The effects of FRP on the levels of **(E)** IgE, **(F)** LTB4, and **(G)** HIS in the serum of rats. All data are presented as $\bar{X}$ ± SD deviation (n = 6/n = 3). Compared with the Control group, ^###^P < 0.001; compared with the model group, ^*^P < 0.05, ^**^P < 0.01, ^***^P < 0.001.

In the control group, we observed fewer mast cells that were dispersed throughout the dermal layer of the skin tissue. The mast cells exhibited metachromatic purple-stained cytoplasm and occasional degranulation. In the skin tissue of the model group rats, we observed significantly higher number of mast cells, dispersed granules, tissue edema, and widened distance between collagen bundles (P < 0.001). However, FRP intervention significantly reduced the number of mast cells, degranulation in mast cells, and vasodilation in the skin tissues of the CU model rats (P < 0.001, Figures 5A, B).

### 3.7 Effect of FRP on the expression of MCT and EPX

Based on immunohistochemistry, the expression levels of MCT protein were significantly higher in the skin tissue of the model group rats compared to the control group rats (P < 0.001). Furthermore, MCT was located around the interstitial cells and small blood vessels as brownish-yellow particles. Compared with the model group, the expression levels of MCT protein were significantly reduced in the skin tissues of the FRP-H and FRP-L group rats (P < 0.01 and P < 0.05, respectively; Figures 5A, C). Immunohistochemistry results also showed that the expression levels of EPX protein were significantly higher in the skin tissues of the model group rats than in the skin tissues of the normal group rats (P < 0.001), but were significantly reduced in the skin tissues of the FRP-H and FRP-L group rats (P < 0.001, P < 0.01, Figures 5A, D).

### 3.8 Effect of FRP on immune biomarkers in rats

The serum levels of IgE, LTB4, and HIS were significantly higher in the model group rats compared with the control group rats (P < 0.001), but were significantly reduced in the FRP-H and FRP-L group rats compared with the model group rats (P < 0.001, Figures 5E–G).

### 3.9 Effect of FRP on skin metabolites in rats

**FIGURE 6:**
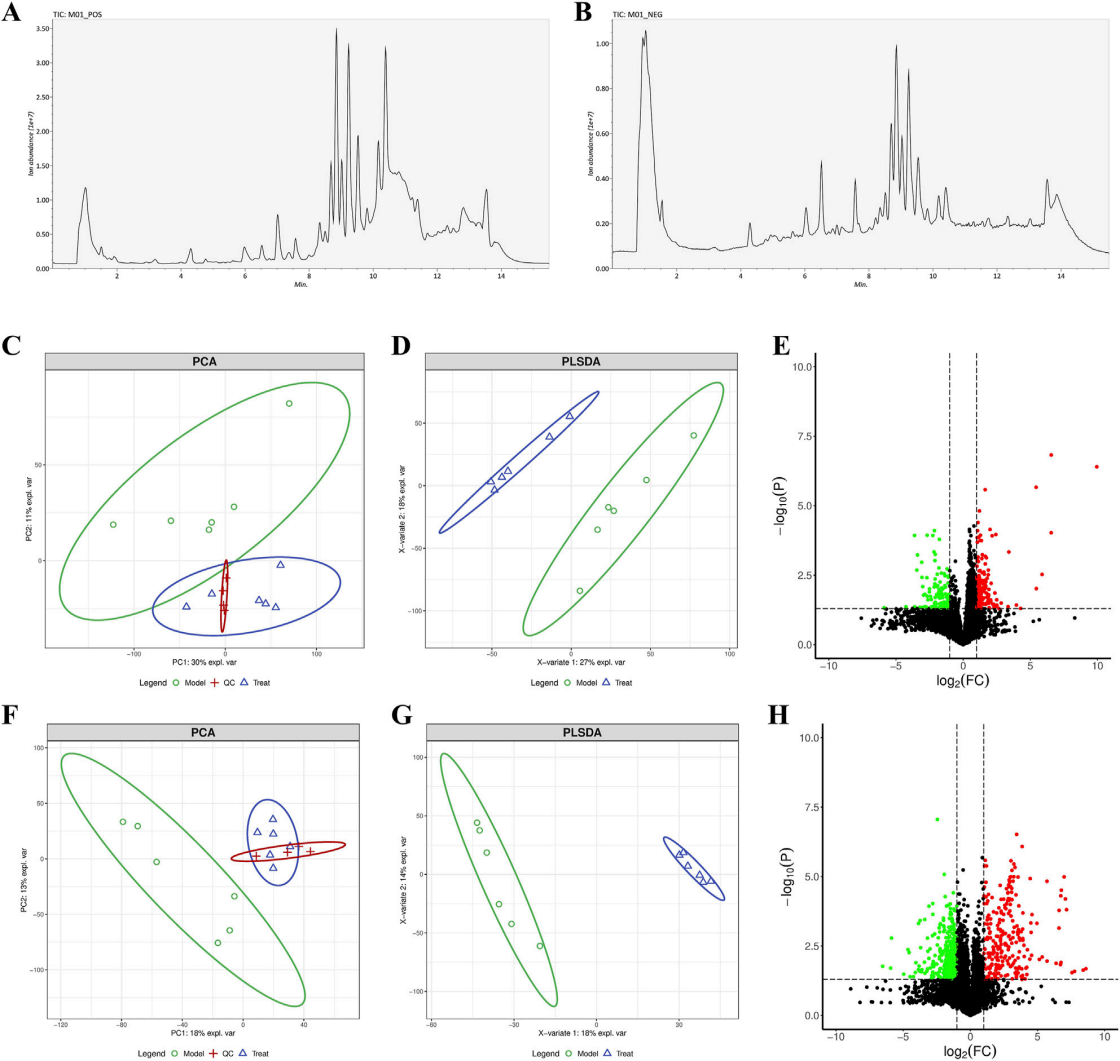
**(A)** Chromatograms of metabolites in positive ion and **(B)** negative ion modes. **(C)** PCA, **(D)** PLSDA, and **(E)** volcano plots under the positive ion mode. **(F)** PCA, **(G)** PLSDA, and **(H)** volcano plots under the negative ion mode.

The characteristic peaks from the LC-QTOF/MS data were extracted and preprocessed. We identified 8,200 chromatographic peaks in the positive ion mode and 8,134 chromatographic peaks in the negative ion mode (Figures 6A, B). This indicated that the instrument was stable and ready for further analysis The PCA and PLSDA results showed that samples from the model group and the FRP treatment group exhibited distinct separation in both positive and negative ion modes. This suggested significant metabolic differences between the model group and the FRP-treatment group rats. Then, we screened metabolites using the OPLS-DA model with VIP value of >1 and P value of <0.05 as threshold parameters and identified 74 differential metabolites (62 upregulated and 12 downregulated) in the positive ion mode and 47 differential metabolites 9 upregulated and 38 downregulated in the negative ion mode (Figures 6C–H).

**FIGURE 7:**
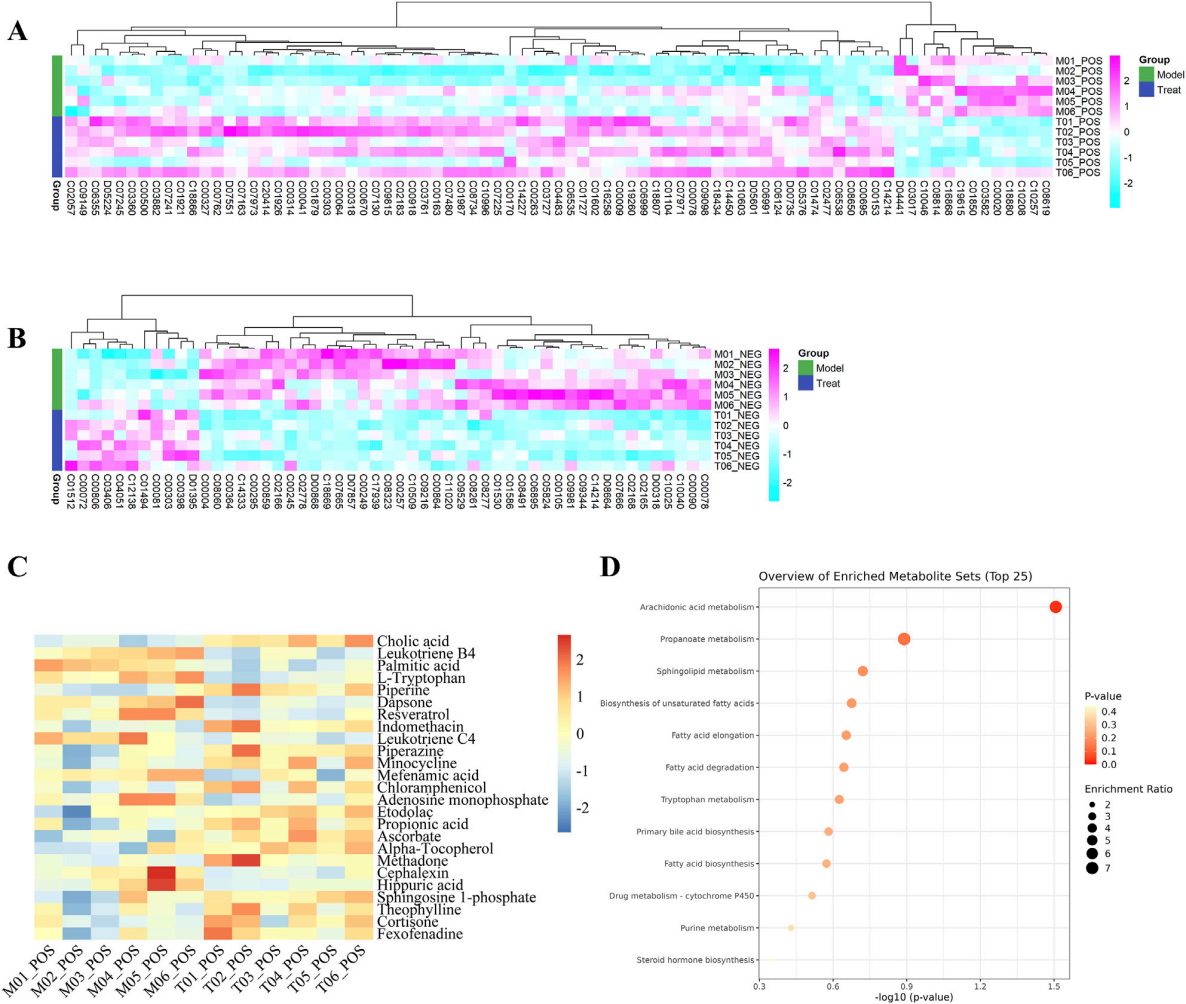
**(A)** Heatmaps of differential metabolites in positive ion and **(B)** negative ion modes. **(C)** Heatmap of differential metabolites and **(D)** Metabolites pathway diagram with an ROC value >0.9. The metabolite levels were indicated with a color code that gradually decreased from purple to blue. The purple color indicated higher levels, whereas blue indicated lower levels.

In the volcano diagram, differential metabolites in the same cluster exhibited similar expression patterns and may have similar functions or participate in the same metabolic processes (Figures 7A, B). The diagnostic value of differential metabolites was verified by plotting ROC curves, we selected differential metabolites with AUC values greater than 0.9 for further analysis. Furthermore, we performed KEGG pathway analysis of the differential metabolites and identified enrichment of pyrimidine metabolism, tryptophan metabolism, arginine biosynthesis, and nicotinate and nicotinamide metabolism (Figures 7C, D).

### 3.10 Results of network pharmacology analysis

**FIGURE 8:**
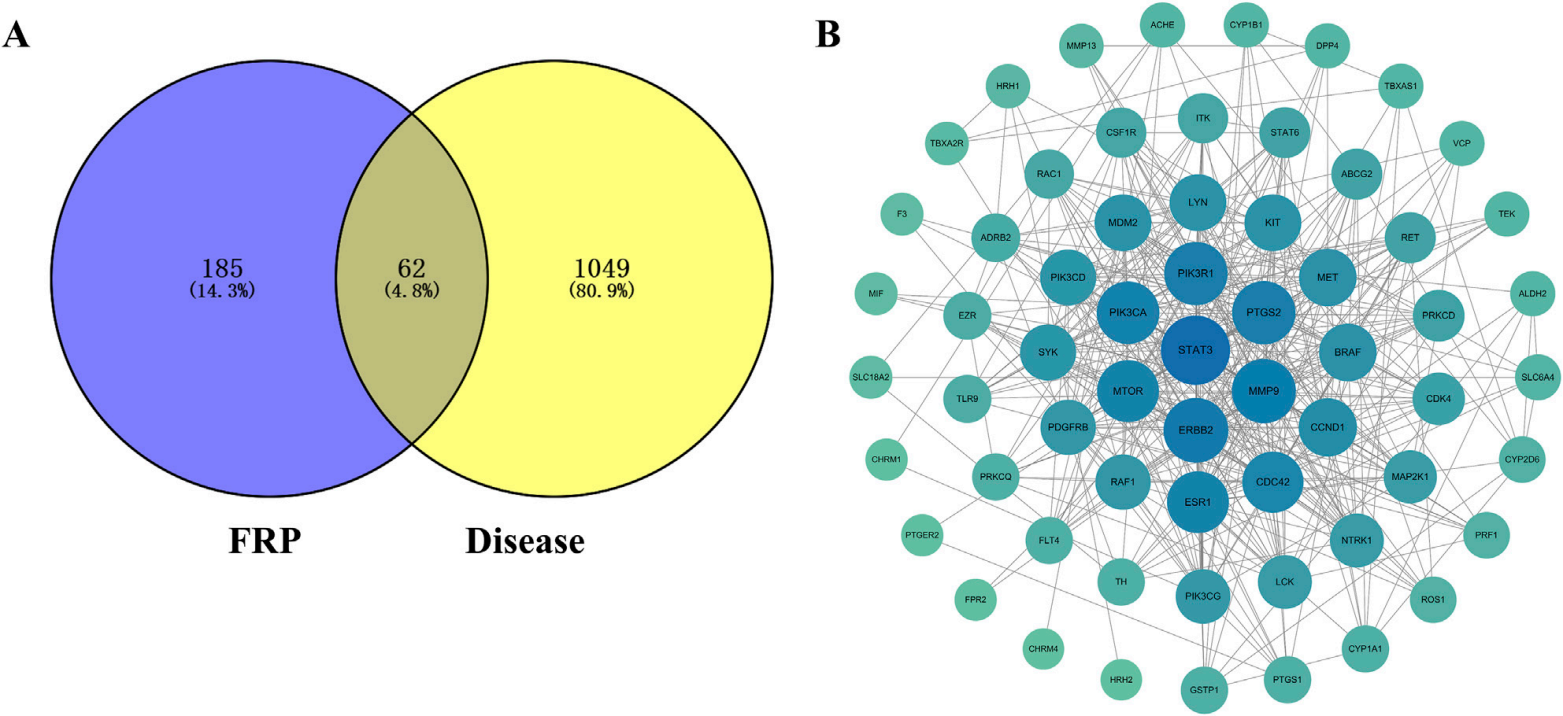
**(A)** Venn diagram of the target points of differential metabolites of FRP and disease target points. **(B)** PPI network diagram of key target points.

We screened FRP chemical components with the top 1% content with SwissADME and identified 10 active ingredients and 189 corresponding targets. We also screened the OMIM, TTD, and Genecards databases for targets corresponding to the disease. After removing duplicates, we obtained 1,111 disease-specific targets. Subsequently, we generated a Venn diagram on the micro-biotech data analysis platform by intersecting 1,111 disease-specific targets with the 189 active ingredient targets (Figure 8).

**FIGURE 9:**
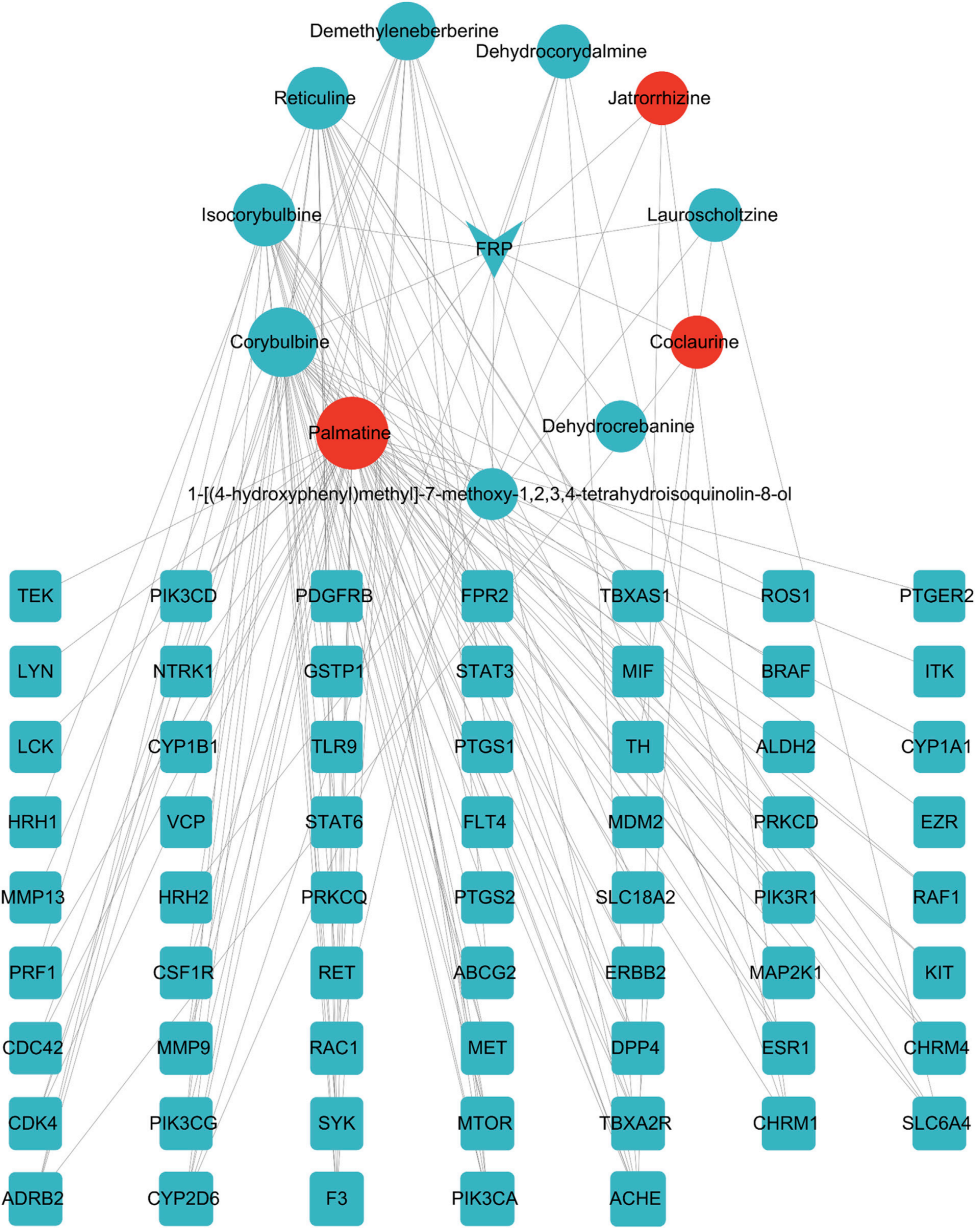
Network diagram of FRP-active ingredients-key target points. The inverted triangle represents the drug, the circles represent components screened by the drug, and the squares represent intersection targets that are color coded from blue to orange based on the degree value. The CytoNCA plug-in was used to analyze the four topological parameters of nodes, including betweenness centrality (BC), closeness centrality (CC), eigenvector centrality (EC), and degree centrality (DC). The orange-red icons were sorted according to the degree value.

The FRP active ingredients and their targets were imported into the String database and a “drug-active ingredient-target” network diagram was constructed using the Cytoscape 3.9.1 software. Based on this analysis, we identified corybulbine, isocorybulbine, reticuline, demethyleneberberine, and dehydrocorydalmine as the main active ingredients of FRP with high degree values (Figure 9).

**FIGURE 10:**
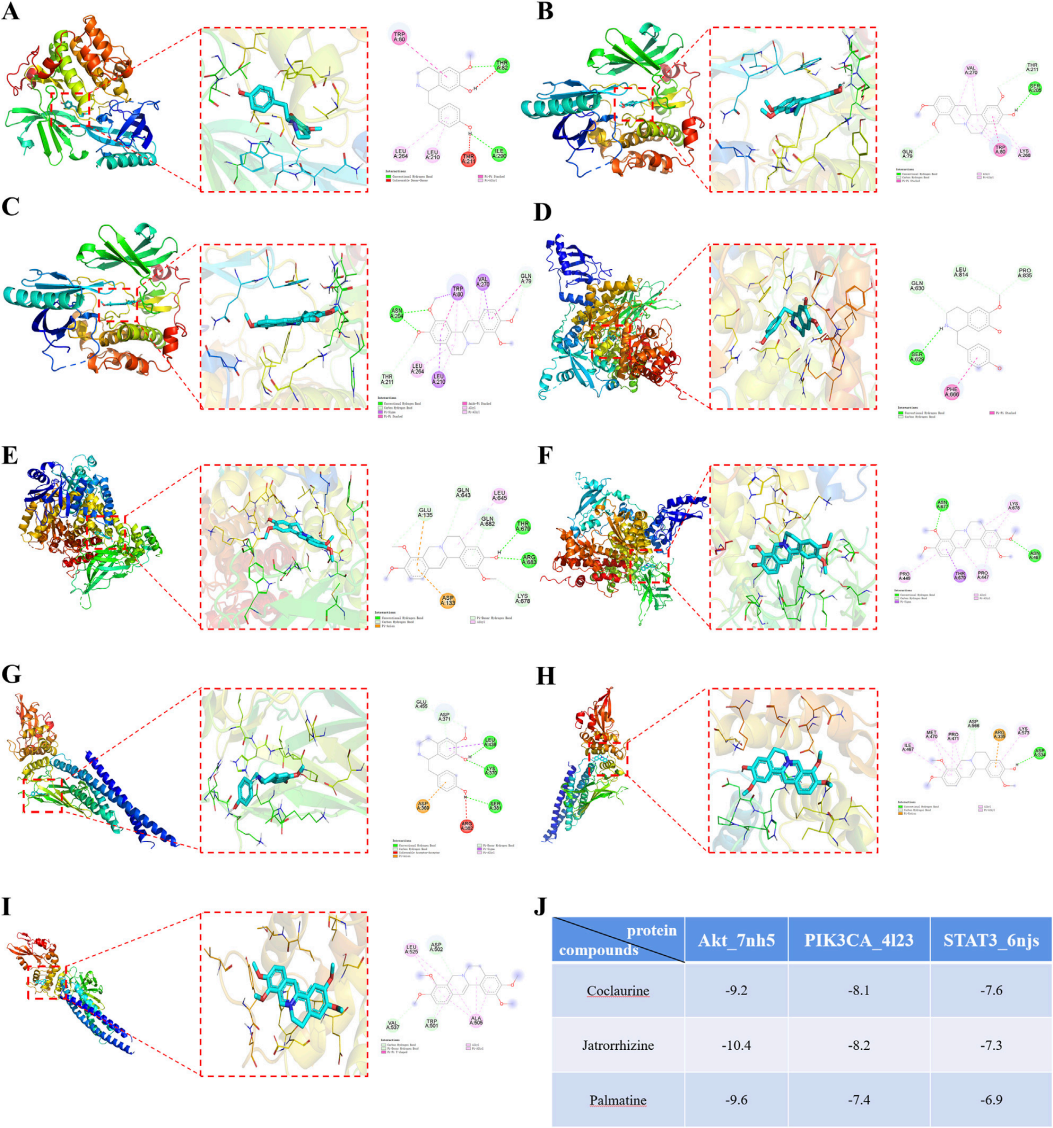
Molecular docking diagrams of **(A)** Akt-Coclaurine, **(B)** Akt-Jatrorrhizine, **(C)** Akt-Palmatine, **(D)** PIK3CA-Coclaurine, **(E)** PIK3CA-Jatrorrhizine, **(F)** PIK3CA-Palmatine, **(G)** STAT3-Coclaurine, **(H)** STAT3-Jatrorrhizine, and **(I)** STAT3-Palmatine. **(J)** The binding energies of the docking complexes in each group.

Molecular docking was performed between the three active ingredients (palmatine, jatrorrhizine, and coclaurine) and the core target. The top three docking structures based on binding energy values were Akt-jatrorrhizine (−10.4 kcal/mol), Akt-palmatine (−9.6 kcal/mol), and Akt-coclaurine (−9.2 kcal/mol) (Figure 10). All the three structures were all less than −7.0 kcal/mol, thereby indicating stable ligand-receptor binding.

**FIGURE 11:**
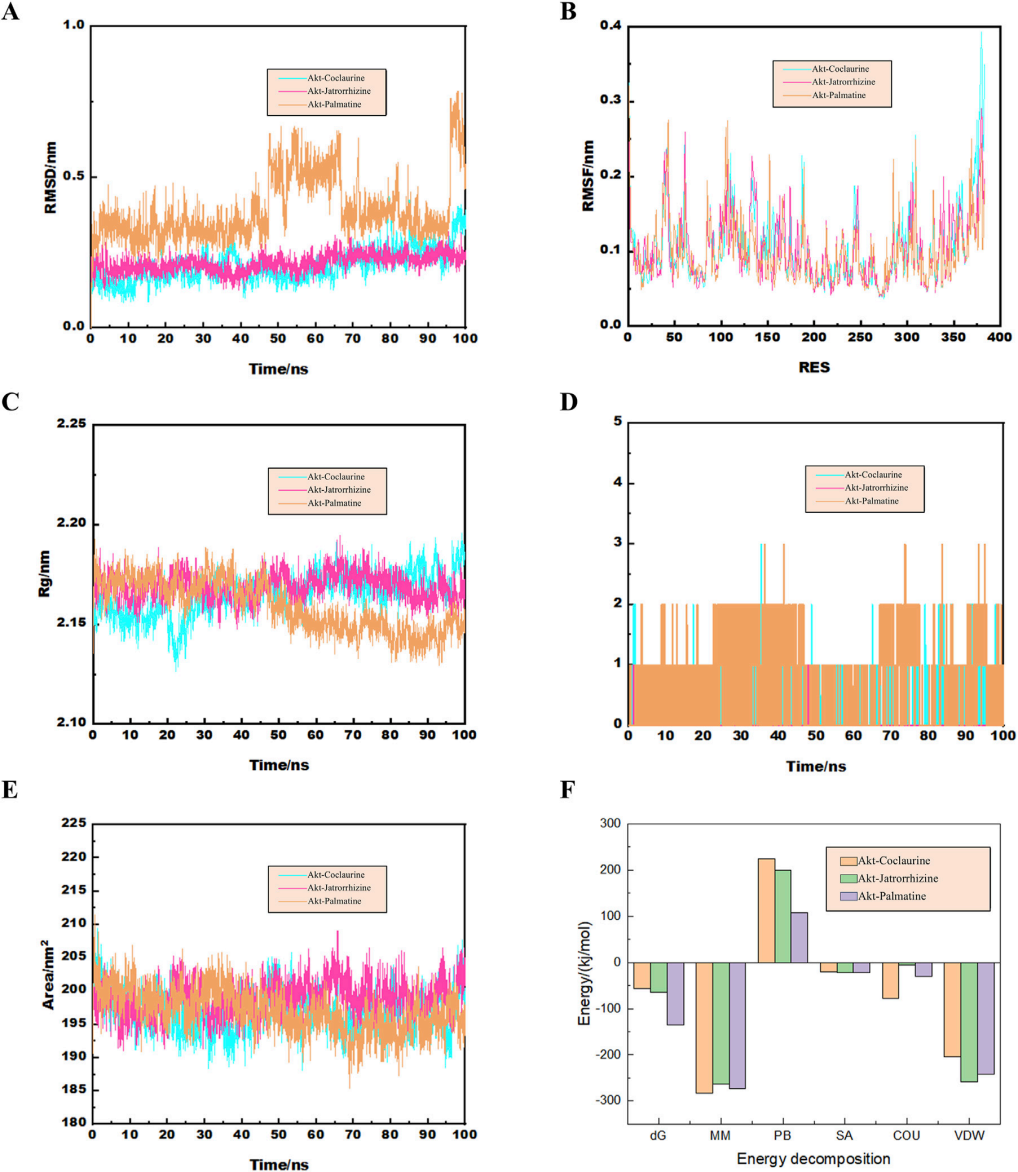
**(A)** The curve diagram of RMSD changes of the docking complex. **(B)** The RMSF analysis diagram of the docking complex. **(C)** The Rg analysis diagram of the docking complex. **(D)** The analysis diagram of the number of hydrogen bonds in the docking complex. **(E)** The curve diagram of the time changes of the solvent and surface area of the docking complex. **(F)** The binding free energy of the docking complex calculated by MMGBSA.

Molecular dynamics simulation results further confirmed the binding of Akt with palmatine, jatrorrhizine, and coclaurine. The Root Mean Square Deviation (RMSD) curve demonstrated that the protein conformation fluctuated during the simulation, but did not break. This indicated that the binding of these three compounds of FRP to Akt was strong and generated a protein structure with good stability (Figure 11A). The Root Mean Square Fluctuation (RMSF) data suggested that the three components did not significantly alter the stability of the binding region of Akt while binding to the small molecules. The RMSF values for most sites in Akt were 0.2 nm, thereby indicating that the structure of the protein-small molecule binding region was stable (Figure 11B). The radius of gyration curve represents the density of the overall protein structure. Jatrorrhizine, coclaurine, and palmatine showed stable gyration radii with the Akt protein from the beginning to the end of the simulation. This suggested that binding of the three compounds did not affect stability of the protein conformation (Figure 11C). The number of hydrogen bonds between Akt and the three compounds are indicative of their binding strength. Palmatine showed the highest hydrogen bond density and strength with Akt, followed by coclaurine and jatrorrhizine (Figure 11D). Furthermore, we also detected the exposure degree of the binding receptor to the surrounding solvent molecules during the simulation. Our data showed that the interaction between the solvent and the surface area of the protein-small molecule complex was stable throughout the simulation process (Figure 11E). The binding of jatrorrhizine and coclaurine molecules to the Akt protein was stable from the beginning to the end of the simulation and the complex structure remained unchanged. The binding of a palmatine molecule to the Akt protein was stable for the first 40 ns, but the stability decreased slowly in the next 60 ns. Overall, the complex remained stable. This suggested reduction in the solvent accessible surface area of the structure. The binding free energy can be used to determine the variability and stability of the ligand-protein binding mode. The Molecular Mechanics Poisson-Boltzmann Surface Area (MMPBSA) binding free energies of the coclaurine, jatrorrhizine, and palmatine molecules were −56.865, −64.71, and −135.327 kJ/mol respectively (Figure 11F).

**FIGURE 12:**
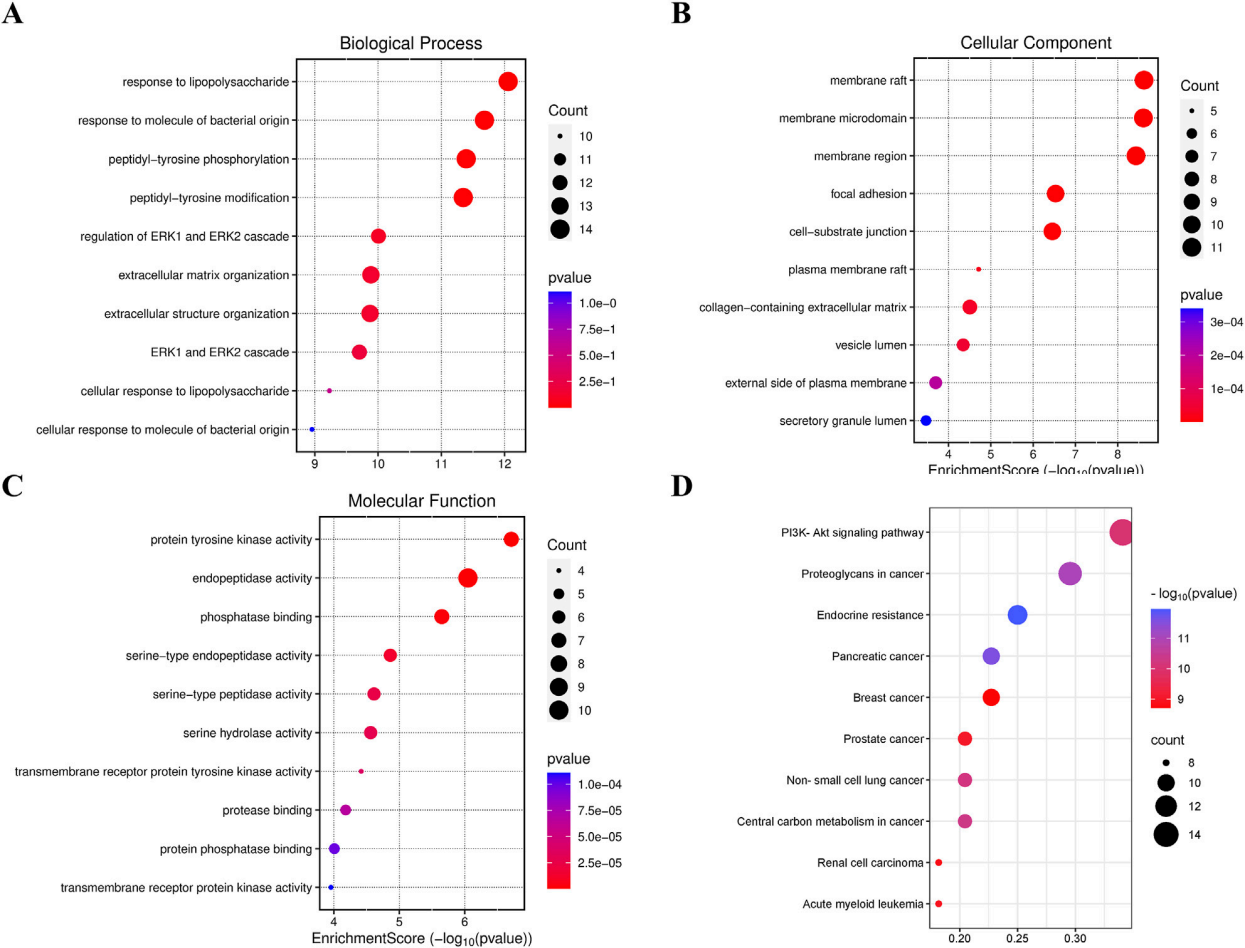
Network pharmacology **(A)** BP, **(B)** CC, **(C)** MF, and **(D)** KEGG enrichment pathway diagrams.

Gene Ontology (GO) and KEGG pathway enrichment analysis of the target proteins was performed using the micro-bioinformation analysis platform. GO analysis included identification of enriched biological processes (BP), molecular functions (MF), and cell components. The top enriched biological processes were cell chemotaxis, regulation of MAP kinase activity, and positive regulation of protein serine/threonine kinase activity. The top enriched cell components were membrane raft, membrane microdomain, and membrane region. The top enriched molecular functions were protein tyrosine kinase activity and protein serine/threonine kinase activity (Figures 12A–C). The top enriched KEGG pathways were Pl3K-Akt signaling pathway, proteoglycans in cancer, endocrine resistance, pancreatic cancer, and breast cancer (Figure 12D).

### 3.11 Combined analysis of network pharmacology and metabolomics

**FIGURE 13:**
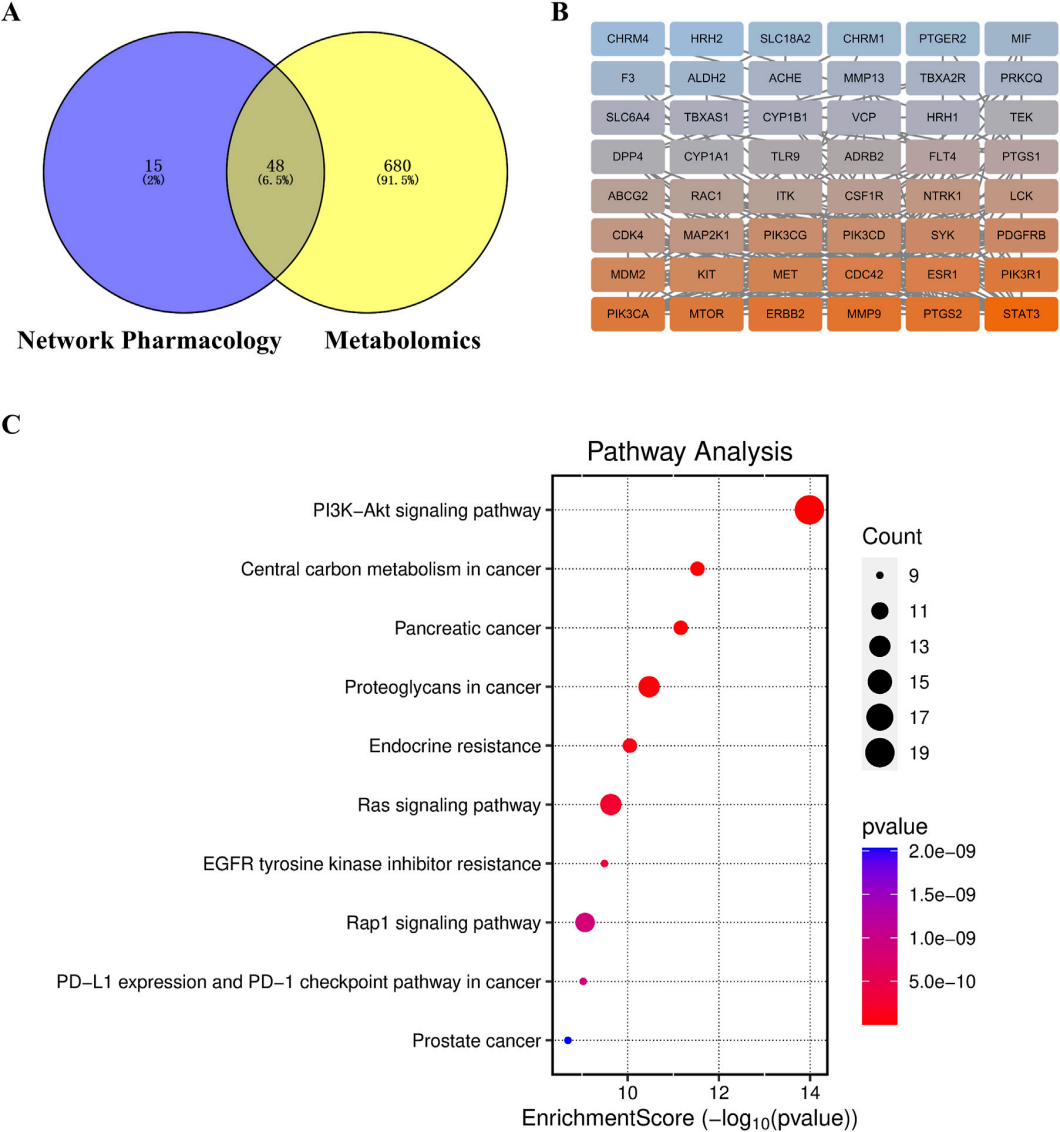
**(A)** Target Venn diagram, **(B)** PPI network diagram of key target points, and **(C)** KEGG enrichment pathway diagram of the combined analysis.

We used the Cytoscape 3.9.1 software and Swiss Target Prediction to predict the potential targets of 58 differential metabolites that were identified by metabolomics. Subsequently, we intersected the 45 targets screened by network pharmacology with the 727 targets related to metabolites and identified 34 common target proteins (Figure 13A). These common target proteins were evaluated using the Cytoscape 3.9.1 software. Based on the degree value, the top target proteins were MMP9, PTGS2, MTOR, ERBB2, CDC42, PIK3R1, and PIK3CA. KEGG pathway analysis of these common target proteins showed that the top enriched pathway were PI3K-Akt signaling pathway, Kaposi sarcoma-associated herpesvirus infection, proteoglycans in cancer, pancreatic cancer, and endocrine resistance (Figures 13B, C).

### 3.12 Effects of FRP on the expression of Akt, p-Akt, PI3K, and p-P13K in rats

**FIGURE 14:**
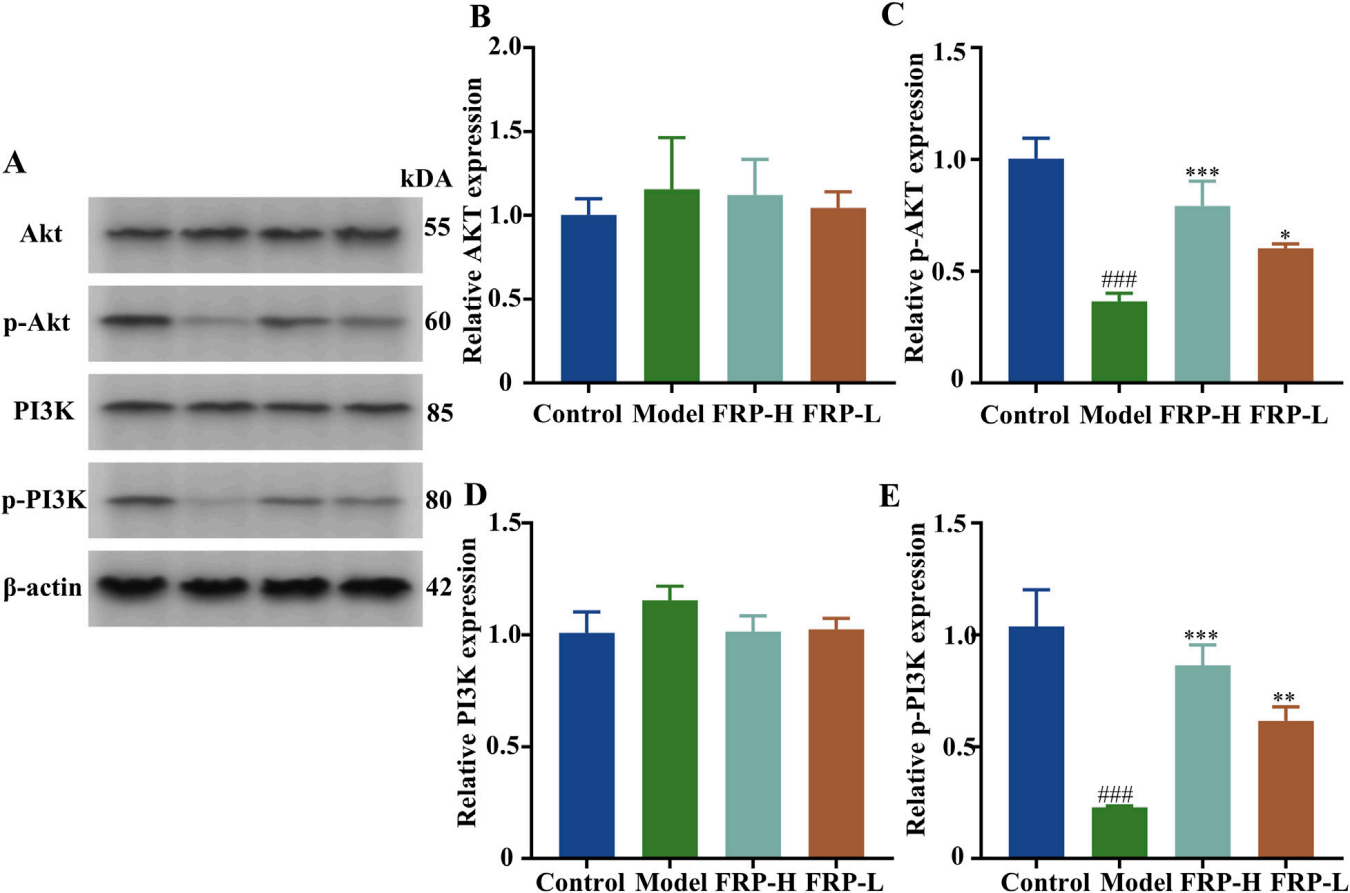
**(A)** Protein band diagrams of each group in the PI3K-Akt pathway. The effects of FRP on the protein expressions of **(B)** Akt, **(C)** p-Akt, **(D)** PI3K, and **(E)** p-PI3K in rats. All data are presented as $\bar{X}$ ± SD deviation (n = 3). Compared with the Control group, ^###^P < 0.001; compared with the model group, ^*^P < 0.05, ^**^P < 0.01, ^***^P < 0.001.

Based on Western blotting, the expression levels of p-Akt and p-P13K were significantly decreased in the model group compared with the control group (P < 0.001). However, the expression levels of p-Akt and p-P13K were significantly increased in the FRP treatment groups compared with the model group (P < 0.001, P < 0.01, P < 0.05, Figure 14).

## 4 Discussion

Recurrent occurrence of itchy wheals or angioedema on the skin surface are the main clinical manifestations of CU. The repeated occurrence of itchy symptoms every day significantly reduces the quality of life in patients with CU (Goncalo et al., 2021; Akca and Tuncer Kara, 2020). FRP is an ethnic medicine that has been used for hundreds of years to clinically treat allergic skin diseases, but its mechanism of action was unclear. Numerous studies have demonstrated that the total alkaloids and other active components of FRP play a significant role in the treatment of Alzheimer’s disease, improvement of hyperglycemia, regulation of cellular apoptosis, and modulation of inflammatory responses. In acute toxicity tests using gavage administration in mice, the total alkaloids of FRP were both safe and effective, thereby indicating significant potential for further clinical development. This study quantitatively evaluated the scratching behavior of CU model and FRP treated CU model rats to determine the efficacy of FRP in alleviating the main symptoms of CU. FRP treatment significantly relieved reduced itching symptoms in the CU model rats. Furthermore, we performed histopathological analysis of skin tissues by H&E staining. The rats were sensitized and challenged with OVA to induce CU-like allergic reactions. H&E staining results showed that low- or high-dose FRP treatment alleviated pathological morphology of skin tissues in the CU model rats to varying degrees.

CU is highly related with IgE-mediated mast cell degranulation and release of histamine (HIS). IgE is an antibody type that binds to mast cells and stimulates them to degranulate and release HIS and other inflammatory mediators upon encountering an allergen (Kolkhir et al., 2024). After patients with CU are first exposed to allergens, the humoral immune response of the body is activated, leading to the production of IgE antibodies. IgE specifically binds to high-affinity receptors on the surface of the mast cells, thereby sensitizing them for future encounter with the same allergen (Orzan et al., 2022; Kolkhir et al., 2022). When the same allergen invades sensitized individuals, it rapidly stimulates effector immune cells to undergo degranulation and triggers release of pro-inflammatory mediators such as HIS and LTB4. HIS acts on the blood vessels of the skin and mucous membranes throughout the body and increases blood vessel permeability and capillary dilation. This leads to formation of wheals and induces clinical symptoms of CU. LTB4, a lipid mediator derived from arachidonic acid, is closely related with the onset of CU (Zeng et al., 2024; Lu et al., 2024). Mast cells recruit effector T cells to the site of inflammation by releasing LTB4. This helps sustain and amplify the inflammatory response. Therefore, LTB4 is a key factor that links activation of mast cells and T cells. It plays an important role in the pathological process of CU and is a major focus of CU treatment research.

Dermal mast cells are main effector cells involved in both wheal formation and sensory nerve stimulation in CU. When mast cells degranulate, they release several pro-inflammatory mediators, including MCT, which are crucial for the development and sustenance of allergic reactions (Puxeddu et al., 2024; Metz et al., 2024). Eosinophils are also regarded as pro-inflammatory cells in the immune response related to CU and release a variety of inflammatory factors and toxic substances. EPX is a toxic protein primarily secreted by eosinophils and is often used as a specific marker for eosinophil activity. The inflammatory response in CU leads to significantly increased levels of MCT and EPX (Elieh-Ali-Komi et al., 2023; Sanchez et al., 2021). Our study results showed that mast cell degranulation was significantly reduced in the CU model rats after FRP intervention. Furthermore, the levels of IgE, HIS, LTB4, EPX, and MCT were significantly higher in the skin tissues of model group rats than those in the control group, but were significantly reduced after FRP intervention. These findings suggested that FRP effectively reduced mast cell degranulation and the release of HIS mediated by IgE.

IL-4, a signature cytokine of the Th2 lymphocytes, promotes proliferation and differentiation of mast cells, and stimulates mast cells to release HIS, a key mediator in the formation of wheals. IL-4 also increases the expression and signal transduction of high-affinity IgE receptor cells. Furthermore, IL-4 regulates IgE expression levels by inducing B cells to switch from IgG synthesis to IgE production, thereby increasing the total serum IgE levels in the body (Wu et al., 2022; Aranda et al., 2023). This induces Th2 lymphocyte-dominated inflammatory response, which is at the core of CU pathogenesis and activation of mast cells. Activated mast cells secrete a variety of inflammatory factors, including IL-4, which further exacerbate CU and the associated vicious cycle (McSweeney et al., 2022). IL-6 activates T cells and exacerbates the inflammatory response in the body (Korn and Hiltensperger, 2021).

IL-12 is a key cytokine that promotes polarization of T cells into the Th1 phenotype. It inhibits secretion of IgE by the B cells. A low concentration of IL-12 often reflects weakened cellular immune response. IL-12 secretion is related to the production of IFN-γ, a representative cytokine of Th1 lymphocytes. IL-12 exerts antagonistic effect on IL-4, a Th2 lymphocyte-related cytokine. IFN-γ inhibits the activity of Th2 cells, promotes differentiation of Th cells into Th1 cells, and blocks the synthesis of IgE (Peng et al., 2021). This study found that FRP treatment effectively restored the normal balance between Th1 and Th2 cells by reducing the levels of pro-inflammatory factors such as IL-4 and IL-6, and increasing the levels of anti-inflammatory factors such as IL-12 and IFN-γ, thereby alleviating the inflammatory response caused by CU.

The metabolomics study identified 58 differential metabolites related to CU and FRP-treated CU model group. Pyroglutamic acid, a natural amino acid derivative, inhibits inflammatory response and improves intestinal health in mice. The state of intestinal health is closely related with the occurrence and development of allergic diseases. Genkwanin, an antioxidant, exerts anti-inflammatory effects by inhibiting inflammatory mediators and related signaling pathways, and protects cells against oxidative stress damage (Gang et al., 2018). Indomethacin, a non-steroidal anti-inflammatory drug, demonstrates antipyretic and anti-inflammatory effects. Cholic acid reduces inflammation by inhibiting the NLRP3 inflammasome (Rashid et al., 2021). Piperazine derivatives are highly effective histamine H1 receptor antagonists and are a valuable therapeutic option for treating CU. The deficiency of argininosuccinate lyase leads to the accumulation of argininosuccinic acid, which is a key factor in altering substances that trigger inflammation. Carbazole derivatives demonstrate significant antibacterial and anti-inflammatory effects. L-Tryptophan plays an important role in regulating inflammatory response in a variety of human chronic inflammatory diseases (Zeng et al., 2024). Isoliquiritigenin inhibits inflammation by suppressing the NF-κB signaling pathway. Piperine is associated with antioxidant activity and reduces inflammation and pain (Choi et al., 2020).

Activation of the PI3K-AKt signaling pathway regulates pyrimidine metabolism, improves mitochondrial morphology, reduces oxidative stress damage, and mitigates the inflammatory response (Shang et al., 2021). Tryptophan, an essential amino acid for the human body, plays a key role in several physiological processes and signal transduction. Tryptophan metabolic pathway regulates a variety of physiological functions, including inflammation. Arginine biosynthesis plays a key role during allergic reactions (Sun et al., 2023). Inflammatory mediators such as HIS, platelet-activating factor, and LT released by the mast cells, stimulate endothelial cells to convert arginine into NO, thereby exacerbating the allergic reaction. Niacin and nicotinamide metabolism regulate inflammation and immune functions (Gonzalez et al., 2023). The changes in the activity and/or levels of these compounds and the functional characteristics of the enriched pathways suggest that the intervention effect of FRP on CU is related to its inflammatory effects.

Metabolomics data can be used to identify metabolome changes induced by FRP treatment that are associated with improving CU, but cannot comprehensively explain the underlying regulatory mechanism. Network pharmacology can be used for in-depth investigation of the mechanism of action of FRP and overcome the drawback of metabolomics. By combining FRP metabolomics and analysis of drug characteristics, we identified jatrorrhizin, coclaurine, and palmatine, all of which are berberine-like compounds, as active ingredients of FRP. These compounds are associated with significant analgesic, anti-inflammatory, and anti-hepatitis effects (Hong et al., 2023). Jatrorrhizine, a key component in FRP, demonstrates significant anti-inflammatory effects. Coclaurine demonstrates good binding energy in several molecular docking studies with various cellular proteins and participates in several cellular processes, including cell differentiation, growth, movement, and apoptosis (Hu et al., 2024). Palmatine is an effective immunomodulatory compound that enhances innate immune response by activating key immune genes. The anti-inflammatory effects of palmatine have been confirmed in several studies. For example, palmatine exhibits synergistic anti-inflammatory effects in LPS-induced microglia and significantly inhibits the production of TNF-α and iNOS. Preliminary studies by our research group have shown that palmatine significantly alleviates CU by regulating inflammation (Wang H. et al., 2024). Jatrorrhizin, coclaurine, and palmatine are all associated with anti-inflammatory properties and are potential active components in FRP involved in the alleviation of CU. Therefore, we further investigated the mechanisms by which these components in FRP alleviate CU.

The combined analysis of network pharmacology and metabolomics data showed that FRP alleviated CU through its regulatory effects on the PI3K/Akt signaling pathway and targets such as STAT3, ERBB2, MMP9, PTGS2, PIK3R1, PIK3CA, and MTOR. High expression of STAT3 in mast cells triggers inflammatory reactions. Plasma levels of MMP9 are significantly increased in the CU patients (Bartko et al., 2024). PTGS2 is a potential regulator of immune responses and inflammation in CU. PIK3R1, a regulatory subunit of PI3K, mediates inflammation and indirectly regulates its own expression by inhibiting the production of IL-6. PIK3CA inhibition is associated with anti-inflammatory effects (Du et al., 2024). MTOR plays a key role in recognizing nutritional signals and modulating cell growth and proliferation. Inhibition of mTOR reduces the levels of inflammatory cytokines. ERBB2 activates the PI3K/Akt signaling pathway and inhibits the expression of inflammatory factors. Our data showed that FRP suppressed CU by modulating the expression of these key targets through regulation of the PI3K/Akt signaling pathway. PI3K/Akt signaling pathway regulates multiple biological processes such as cell division, proliferation, apoptosis, and metabolism, and plays an important role in the inflammatory response (Hu et al., 2023). Several studies have shown that the PI3K/Akt signaling pathway is involved in relieving the symptoms of CU by negatively regulating the production of IgE, thereby suppressing the activation and degranulation of mast cells. Molecular docking and molecular dynamics simulation results showed stable binding of Akt to the three active components of FRP, namely, palmatine, jatrorrhizine, and coclaurine. Furthermore, we verified that the expression levels of p-PI3K and p-Akt were significantly higher in the FRP group than in the model group. This strongly suggested that FRP inhibited the activation of mast cells and susbequent release of inflammatory mediators by regulating the PI3K/Akt signaling pathway.

## 5 Conclusion

Based on integrated analysis of metabolomics and network pharmacology data, and evaluation of the expression of immune-related indicators, we observed that FRP effectively alleviated the clinical symptoms of CU by reducing inflammation through modulation of the PI3K/Akt signaling pathway. Higher concentration of FRP demonstrated increased activation of the PI3K/Akt signaling pathway and significant downregulation of inflammatory factors. Concurrently, the frequency of scratching was reduced. These findings suggested a positive correlation between FRP concentration, AKT pathway modulation, and downstream biological effects. Based on our results in this study, we plan to conduct clinical trials to further obtain more comprehensive and reliable data regarding the efficacy and safety of FRP to develop robust evidence for supporting its broad application for the clinical management of urticaria.

## Data Availability

The raw data supporting the conclusion of this article will be made available by the authors on request. The metabolomics data presented in the study are openly available in Metabolights at accession number MTBLS9724.
